# Ergonomic Assessment of Robotic versus Thoracoscopic Thymectomy

**DOI:** 10.3390/jcm13071841

**Published:** 2024-03-22

**Authors:** Riccardo Taje, Michael Peer, Filippo Tommaso Gallina, Vincenzo Ambrogi, Azzam Sharbel, Enrico Melis, Stefano Elia, Matot Idit, Francesco Facciolo, Alexandro Patirelis, Roberto Sorge, Eugenio Pompeo

**Affiliations:** 1 Department of Thoracic Surgery, University of Rome “Tor Vergata”, 00133 Rome, Italy; r.taje@virgilio.it (R.T.); ambrogi@uniroma2.it (V.A.); alexandro.patirelis@hotmail.it (A.P.); 2 Doctoral School of Microbiology, Immunology, Infectious Diseases and Transplants, MIMIT, University of Rome “Tor Vergata”, 00133 Rome, Italy; 3 Department of Thoracic Surgery, Ichilov Medical Center, Tel Aviv 6423906, Israel; michaelp@asaf.health.gov.il (M.P.); sharbelazzam8@hotmail.com (A.S.); 4 Department of Thoracic Surgery, IRCCS National Cancer Institute Regina Elena, 00144 Rome, Italy; filippogallina92@gmail.com (F.T.G.); enrico.melis@ifo.it (E.M.); francesco.facciolo@ifo.it (F.F.); 5 Department of Medicine and Health Sciences “V. Tiberio”, University of Molise, 86100 Campobasso, Italy; stefano.elia@unimol.it; 6 Department of Anaesthesia and Intensive Care, Ichilov Medical Center, Tel Aviv 6423906, Israel; iditm@tlvmc.gov.il; 7 Department of Biostatistics, University of Rome “Tor Vergata”, 00133 Rome, Italy; sorge@uniroma2.it

**Keywords:** complete thymectomy, robotic thymectomy, VATS thymectomy, robotic surgery, thoracoscopy, ergonomic, thymoma

## Abstract

**Introduction**: Robotic and thoracoscopic surgery are being increasingly adopted as minimally invasive alternatives to open sternotomy for complete thymectomy. The superior maneuverability range and three-dimensional magnified vision are potential ergonomical advantages of robotic surgery. To compare the ergonomic characteristics of robotic versus thoracoscopic thymectomy, a previously developed scoring system based on impartial findings was employed. The relationship between ergonomic scores and perioperative endpoints was also analyzed. **Methods**: Perioperative data of patients undergoing robotic or thoracoscopic complete thymectomy between January 2014 and December 2022 at three institutions were retrospectively retrieved. Surgical procedures were divided into four standardized surgical steps: lower-horns, upper-horns, thymic veins and peri-thymic fat dissection. Three ergonomic domains including maneuverability, exposure and instrumentation were scored as excellent(score-3), satisfactory(score-2) and unsatisfactory(score-1) by three independent reviewers. Propensity score matching (2:1) was performed, including anterior mediastinal tumors only. The primary endpoint was the total maneuverability score. Secondary endpoints included the other ergonomic domain scores, intraoperative adverse events, conversion to sternotomy, operative time, post-operative complications and residual disease. **Results**: A total of 68 robotic and 34 thoracoscopic thymectomies were included after propensity score matching. The robotic group had a higher total maneuverability score (*p* = 0.039), particularly in the peri-thymic fat dissection (*p* = 0.003) and peri-thymic fat exposure score (*p* = 0.027). Moreover, the robotic group had lower intraoperative adverse events (*p* = 0.02). No differences were found in residual disease. **Conclusions**: Robotic thymectomy has shown better ergonomic maneuverability compared to thoracoscopy, leading to fewer intraoperative adverse events and comparable early oncological results.

## 1. Introduction

Thoracoscopic and robotic complete thymectomy are being increasingly adopted as minimally invasive surgical alternatives to open sternotomy for the treatment of thymic tumors and other disorders [1,2,3].

Since the initial reports, robotic surgery has been deemed particularly fit for surgical procedures in the anterior mediastinal space due to the three-dimensional magnified vision and the enhanced degrees of freedom offered by robotic arms [4,5,6].

As compared to thoracoscopy, several reports demonstrated lower conversion rate, minor intraoperative blood loss and milder post-operative complications, as well as lower residual disease rate following robotic thymectomy [7,8]. However, a recent meta-analysis failed to demonstrate clear benefits of robotic versus thoracoscopic thymectomy [9]. Thus, high-quality evidence favoring one approach over the other is still lacking. 

From an oncological point of view, no difference could be found in the overall or disease-free survival between robotic or thoracoscopic complete thymectomy [3,4,10]. However, most of the reported studies entailing either robotic or thoracoscopic thymectomy included highly selected patients with small thymomas at low risk of perioperative complications or residual disease [11]. Thus, both a perioperative and oncological outcome comparison may be hindered by strict patient selection criteria. More recently, both robotic and thoracoscopic surgery are being considered reliable, even for resecting large thymomas or performing more technically demanding surgical procedures. This novel perspective may bring back the focus on the ergonomic differences between robotic and thoracoscopic surgery.

In order to better address the differences between robotic and thoracoscopic thymectomy, we retrospectively compared these two minimally invasive techniques under an ergonomic perspective. We adapted a previously developed ergonomic scoring system based on intraoperative impartial findings that are easily recognizable and quantifiable [12]. In addition, the relationship between ergonomic-based scores and operative time, as well as perioperative endpoints, was tested.

## 2. Materials and Methods

In this retrospective, multicentric study, clinical, surgical and perioperative data of patients undergoing either robotic or thoracoscopic complete thymectomy in the period between January 2014 and December 2022 at three Mediterranean institutions were retrospectively analyzed (ethical board approval number: 1465/21 Approval Date: 23 February 2021). Written informed consent for the surgical procedure was obtained from all patients. Clinical charts and surgical recordings were collected and analyzed according to a previously adopted scoring system. Requirements for additional ports, conversion to sternotomy, surgical duration, intraoperative adverse events and data regarding the postoperative recovery and pathological report were also compared.

### 2.1. Scoring System 

Recordings of the surgical procedures were anonymously collected and numbered for subsequent ergonomic assessment. Three thoracic surgeons, chosen among the authors (RT, EP and TG), independently reviewed and rated the video recordings. Each investigator was blinded to the scoring data retrieved from the other surgeon. A previously used scoring system (3 = excellent, 2 = satisfactory and 1 = unsatisfactory) was adapted for complete thymectomy in the assessment of three ergonomic domains: maneuvering, exposure and instrumentation, as summarized in Table 1 [12]. Evaluation and scoring of each ergonomic domain were applied to four standardized surgical steps: dissection of lower thymic horns, upper thymic horns, innominate vein and perithymic fat. Each step was individually evaluated and scored, and each score was summed up to obtain the total for each ergonomic domain. Before the initiation of the analysis, a brief pilot-training program was carried out by allowing the reviewers to score selected video recordings of robotic (3 videos) or thoracoscopic (3 videos) thymectomy in order to get confidence with the scoring system.

### 2.2. Endpoint Measures 

The primary endpoint of the study was the ergonomic maneuvering domain total score. Secondary endpoints were the ergonomic exposure and instrumentation domain total score, the three ergonomic domain scores measured for each surgical step, intraoperative adverse events, need for additional ports, conversion to sternotomy, operative time, 30-day mortality and 30-day morbidity rates. In the patients with pathologic confirmation of thymic epithelial tumor, the presence of residual microscopic or macroscopic disease was also considered as a secondary endpoint. Finally, the relationship between the ergonomic maneuvering domain total score and the other operative variables were also analyzed.

Intraoperative adverse events were defined as any event requiring additional care compared to the normal progress of the surgical procedures. Intraoperative adverse events were divided into two groups: major that needed additional active management, including bleeding that required additional surgical maneuvering to achieve hemostasis, the need of additional ports or conversion to median sternotomy or thoracotomy, and minor that were treated conservatively, including minor bleeding that stopped spontaneously. In the event of conversion, ergonomic evaluation was performed until the event that led to conversion. Operative time was calculated in minutes from skin incision to skin closure.

### 2.3. Patients’ Selection

Clinical charts, surgical reports and recording were retrieved and extensively reviewed for included patients undergoing either robotic or thoracoscopic complete thymectomy due to early-stage anterior mediastinal tumors. Patients with non-thymomatous myasthenia gravis were also enrolled. Patients undergoing partial thymectomies, subxiphoid or transcervical approaches were excluded. Only patients with complete clinical and operative data regarding the surgical approach and the perioperative progress, with age higher than 18 years old, an American Society of Anesthesia (ASA) score ≤ 2 and body mass index (BMI) between 18 and 28 were enrolled. Exclusion criteria were advanced stage at presentation defined as Masaoka-Koga III and IV, as well as TNM ≥ T3N0M0 stages, patients with clear signs of extensive or calcified pleural adhesions, previous clinical history of pleurodesis or surgical procedures in the targeted hemithorax or involving the mediastinum, as well as history of thoracic radiation therapy or previous chemotherapy for intrathoracic neoplasms.

Preoperatively, all the patients underwent clinical and neurologic examination, routine blood tests and pulmonary as well as cardiovascular function tests. Chest contrast-enhanced computed tomography and positron emission tomography were performed for clinical staging. The operative risk has been assessed by the ASA score.

### 2.4. Surgical Procedure

Regardless of the surgical approach, all the patients were positioned supine with the operated hemithorax elevated to 30°. The homolateral shoulder remains flat in order to avoid impingement with the robotic or thoracoscopic instrumentations. Surgical laterality was decided by taking into account both tumor localization and individual preference of the surgeon.

In most instances, robotic and thoracoscopic thymectomy were achieved through three centimetric ports. A 12 mm camera port for a 30-degree camera was positioned at the fifth intercostal space along the anterior axillary line. The 8 mm operative ports were positioned at the fifth intercostal space along the midclavicular line and at the third intercostal space laterally to the pectoralis major muscle. In the thoracoscopic group, as an alternative to the three ports approach, a single 3 cm incision at the fifth intercostal space with an additional centimetric incision at the eight intercostal space could be performed. In both robotic and thoracoscopic thymectomy, the use of carbon dioxide (CO_2_) insufflation was decided according to the surgeon preference and expertise. In the case of CO_2_ insufflation, maximal flow of 5 mL/h and maximal CO_2_ pressure 7 mmHg were used.

Complete thymectomy was defined as en bloc removal of the gland, including the thymic and adipose tissue, and of the tumor within the phrenic nerves (laterally), diaphragm (inferiorly) and the thyro-thymic ligaments and innominate vein (superiorly) [13]. All the surgical procedures were performed by experienced surgeons in high-volume centers specialized in pulmonary and mediastinal oncological minimally invasive surgery. In all the three centers involved in the analysis, both robotic and thoracoscopic technical facilities were widely available with no limitations for the thoracic unit.

### 2.5. Pathological Assessment 

All the specimens were analyzed by an experienced pathologist and classified by the WHO Histological Classification of Thymomas. Tumor invasion was assessed both by TNM classification of malignant thymic tumors and the Masaoka–Koga staging system. According to previous reports, Masaoka–Koga stage I and II as well as T1, T2 and N0, M0 were defined as early-stage thymomas and included in the study. Complete resection (R0) was defined as no evidence of residual tumor tissue. Incomplete resection was defined as evidence of microscopic (R1) or macroscopic (R2) residual tumor tissue.

### 2.6. Video Analysis

Videos were analyzed only during active movement of robotic or thoracoscopic instruments. Videos were reviewed from the first mediastinal pleura incision to complete the thymic dissection. Trocar positioning, specimen retrieval as well as chest tube positioning and skin closure have not been reviewed. During video analysis, video reproduction could be sped up to 5× to allow for sensitive scoring of each ergonomic domain. When an ergonomic score of less than 3, according to the previously presented scoring system, was identified, the video fragment was slowed down to the baseline speed. The video section was then reviewed and scored. In the case of sustained pauses of the surgical instrument movement, the video could be skipped until active movement restarted. Video reviews were conducted anonymously as the reviewers could not identify the patient and the institution performing the surgical procedure. An exemplifying frame of the video analysis method and scoring system is shown in Video S1 in the Supplementary Material.

### 2.7. Statistical Analysis 

All data were initially entered into an Excel database (Microsoft, Redmond, WA, USA) and the analysis was performed using IBM Corp.2017. IBM SPSS Statistics for Windows, Vers.25.0. Armonk, NY, USA: IBM Corp.

Descriptive statistics consisted of the mean ± standard deviation for parameters with normal distributions (after confirmation with histograms and the Kolgomorov–Smirnov test), median and interquartile range (first quartile; third quartile) for variables with non-normal distributions.

Following a crude analysis of the entire cohort, 1:2 propensity score matching with age, sex, BMI, and tumor diameter as covariates was performed among patients with anterior mediastinal tumors undergoing three-port robotic or thoracoscopic complete thymectomy; adequate video recordings were available to create two homogeneous cohorts: an experimental robotic group including 68 patients and a control thoracoscopic group including 34 patients.

For each group, the standardized mean difference, range and 95% confidence interval were calculated, and the homogeneity among the two groups was tested by one way analysis of variance using the same covariates. Comparison among groups was performed with the ANOVA one-way for normal variables or the chi-squared test or Fisher’s exact test (if cells < 5) for frequency variables, and Kruskal–Wallis (groups > 2) or Mann–Whitney (groups = 2) for non-normal variables. A *p*-value < 0.05 was considered statistically significant.

The relationship between variables was tested by the Pearson R correlation analysis. The consistency and reliability of interobserver scores were assessed by Cronbach’s Alpha in 30 patients (20 in the robotic group and 10 in the thoracoscopic group).

## 3. Results

A total of 219 patients’ data were retrieved for retrospective analysis from the three participating institutions. Demographic and baseline data are presented in Table 2. The patient selection process is depicted in Figure S2. Complete thymectomy was performed by thoracoscopy in 70 patients and by a robotic approach in 149 patients. Pathological results and surgical laterality in the entire population are depicted in Table 2. Results of the perioperative and ergonomic analysis of the entire population are presented as Supplementary Materials. The thymectomy was performed due to an anterior mediastinal tumor in 145 patients (66.2%).

Propensity score matched analysis was performed in the anterior mediastinal tumor cohort to create two groups of 68 and 34 patients undergoing robotic and thoracoscopic complete thymectomy, respectively, which proved well-matched for baseline data and tumor characteristics, as shown in Figure S3. Baseline and surgical characteristics of the post-propensity matched population are presented in Table 2.

### 3.1. Perioperative Data Comparison 

**Figure 1:**
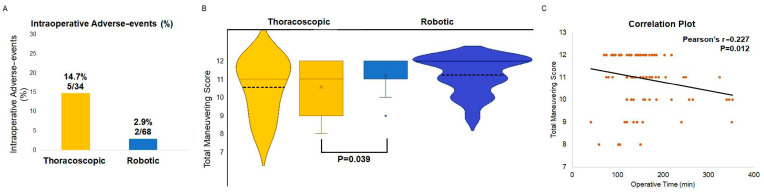
(**A**) Intraoperative adverse-events distribution rate in the propensity score matched population with anterior mediastinal tumors showing lower adverse-events in the robotic group. (**B**) Total maneuvering score in the propensity score matched population with anterior mediastinal tumors showing higher scores in the robotic group. (**C**) Correlation plot showing an inverse correlation between operative time and total maneuvering score in the propensity score matched anterior mediastinal tumors cohort.

At intergroup comparison (robotic group versus thoracoscopic group), as shown in Figure 1A, robotic group had significantly lower intraoperative adverse events compared to the thoracoscopic group (*p* = 0.02). Major adverse events were recorded in 1 (1.5%) versus 2 patients (5.9%) while minor adverse events were recorded in 1 (1.5%) versus 3 patients (8.8%), respectively. Major adverse events were due to bleeding from the innominate vein (one patient in both the robotic and the thoracoscopic group) or internal thoracic vein (one patient in the thoracoscopic group), respectively. Particularly, innominate vein injury required conversion to sternotomy in both instances, while internal thoracic vein bleeding was controlled with surgical clips positioning following conversion from single port to biportal thoracoscopy. There was no conversion to thoracotomy. Minor adverse events included 4 minor bleedings treated by conservatively in one patient and both by hemostatic patches and conservatively in 3 patients.

No difference could be found in the need for additional ports (1/68, 1.5% vs. 2/34, 5.9%, *p* = 0.6), conversion to sternotomy (1/68, 1.5% vs. 1/34, 2.9%, *p* = 0.8) and operative time (*p* = 0.68). Carbon Dioxide was used in 27/68, 39.7% patients in the robotic group and in 9/34, 26.5% patients in the thoracoscopic group (*p* = 0.27). There was no operative mortality whereas post-operative complications were similar between the two groups as depicted in Table 2 (*p* = 0.55). None of the post-operative complications required reoperation while additional pharmacological therapy was necessary in 2 patients in the robotic group and in one patient in the thoracoscopic group (Clavien-Dindo grade II). The other postoperative complications described in Table 2 resolved conservatively (Clavien-Dindo grade I).

As far as the oncological outcomes was regarded, there was no difference in residual disease between the robotic group compared to the thoracoscopic group (7/59, 11.7% vs. 3/19, 15.8%, *p* = 0.65).

### 3.2. Ergonomic Analysis 

Results of the ergonomic analysis are detailed in Figure 1B and in Table 3. As far as the total score of the three domains were taken into account, only the maneuvering score was better in the robotic group (*p* = 0.0039) as depicted in Figure 1B whereas no differences could be found between thoracoscopic and robotic thymectomy according to the total exposure (*p* = 0.943) and instrumentation scores (*p* = 0.970). Particularly, both maneuvering and exposure domains scores were higher in the robotic group in the perithymic fat dissection surgical step (*p* = 0.003, *p* = 0.027). No difference could be found between robotic and thoracoscopic thymectomy in the other ergonomic domains in any surgical step. Maneuvering total score (Figure 1C) was significantly and inversely correlated to operative time (rho= −0.227; *p* = 0.012). Results of the ergonomic analysis for the entire population is presented in Supplementary Table S1 and Figure S3.

### 3.3. Intergroup Agreement

As far as the inter-rater reliability was concerned, overall, the Cronbach Alpha for the maneuvering, exposure and instrumentation domains was 0.84, 0.94, and 0.89, respectively, indicating a satisfactory coherence of scoring amongst the three reviewers.

## 4. Discussion

Robotic surgery holds promise as a reliable approach for thymectomy, although this perception is not yet supported by evidence-based data [2,7,9]. This is the first study aimed at comparing thoracoscopic versus robotic complete thymectomy from an ergonomic perspective in a propensity score matched population undergoing complete thymectomy for anterior mediastinal tumors. Our results have shown significantly higher scores in the total maneuverability domain in the robotic group. In particular, during perithymic fat dissection, the robotic group showed a higher score in both maneuverability and exposure to ergonomic domains.

Our analysis has been conducted in a propensity score matched cohort limited to patients with anterior mediastinal tumors. In fact, because of tumor encumbrance in a limited surgical field, such as the anterior mediastinum, this population has been deemed particularly sensitive to an assessment of surgical ergonomic domains.

Our results seem to objectify the assumption that the robotic enhanced three-dimensional magnified vision, as well as the wider instruments range of motion, may improve the perception of depth and accuracy of surgical maneuvering in the anterior mediastinal space. Conversely, we speculate that two-dimensional thoracoscopic surgery that lacks depth perception can affect hand–eye coordination due to less-accurate judgment of the distance between the endoscopic instruments and target tissue, even though a novel 4K high-definition video system may contribute to minimize this limitation [14,15,16,17].

Although robotic surgery was demonstrated to improve maneuverability and exposure, no differences could be retrieved in the instrumentation ergonomic domain. This finding may be related to the surgical technique. Differently from the larger operative field during pulmonary anatomical resections that require relevant repositioning of the camera and of the instruments, thymectomy requires only minimal and stepwise adjustments limiting fencing or crowding. Thus, the narrower operative field may justify the low variability in the instrumentation ergonomic domain that we have found between robotic and thoracoscopic complete thymectomy techniques.

For the study purposes, only patients undergoing a unilateral approach have been included. However, the optimal laterality of the surgical approach in complete thymectomy is still debated. In myasthenia gravis, both right- and left-sided approaches have been employed with satisfactory results [18,19]. The suitability of a certain surgical approach can be maximized in anterior mediastinal tumors where the location of the mass may suggest the optimal surgical laterality. In our analysis, ergonomic analysis failed to differentiate between right- or left-sided surgical approaches, as shown in Table S1. This finding, even if the study was not designed and balanced for this purpose, shed new light on surgical laterality in thymectomy and corroborated a tumor-based, customized approach rather than an a priori left- or right-sided approach. Further study may be necessary to clarify the best laterality in both nonmyasthenic and myasthenic patients presenting at surgery with anterior mediastinal tumors.

**Figure 2:**
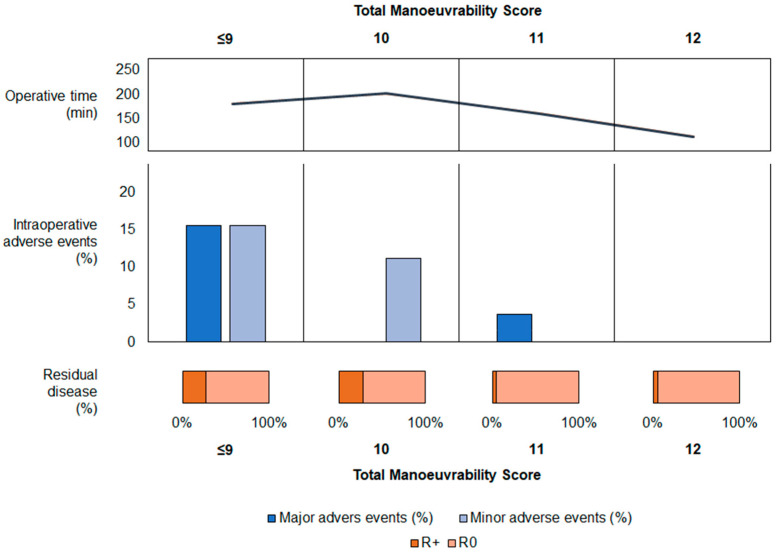
Graphical correlation between the main clinical outcomes and the total maneuvering ergonomic score. Median operative time (**above**), major and minor intraoperative adverse events (**central**) and residual disease (**below**) are presented for each total maneuverability score.

In an attempt to improve the reliability of our scoring system, we correlated total maneuvering score and clinical endpoints (Figure 2). Intraoperative adverse events progressively decreased along with improvement in the total maneuverability score. Moreover, the operative time showed a significant and inverse correlation with total maneuvering score in both entire and propensity score matched populations (Figure 1C and Supplementary Figure S3C).

These results compare with previous findings demonstrating a stepwise reduction in both operative time and intraoperative adverse events along with improvement in dexterity in complete thymectomy [20,21,22] and with improved ergonomic in pulmonary resection [23,24,25].

Indications for robotic or thoracoscopic thymectomy have been safely extended to progressively more demanding procedures including complete thymectomy with vascular reconstruction [26,27]. However, most of the studies focused on highly selected patients with low operative risk. Thus, advantages and disadvantages in the perioperative endpoints are still unclear. This is mainly due to the high heterogeneity among studies in both patient selection criteria and results [9].

Although, in our study, we have found no intergroup difference in morbidity, robotic surgery resulted in lower intraoperative adverse events compared to the thoracoscopic group. Particularly, robotic surgery seems to be associated with fewer minor adverse events, mostly consisting of minor parenchymal bleeding requiring conservative management only, which may contribute to suggesting superior ergonomic accuracy of robotic thymectomy. This result corroborates those of previous reports [28,29]. Conversely, major intraoperative adverse events as well as the conversion rate were similar between groups. This finding is also confirmed by the similar ergonomic scores achieved by the two surgical approaches when dealing with vascular structures.

Finally, we analyzed the relationship between ergonomic characteristics and oncological outcomes. In our analysis, we have found no difference in residual disease between study groups. This finding is consistent with previous reports [2,8].

Regarding thymic epithelial tumors, because of their indolent biological behavior, the disease-free interval is generally deemed the most reliable oncological endpoint [7,8,9]. However, in this case, due to the short follow-up available in the robotic group due to the recent introduction of the technique, residual disease only has been adopted as an adequate early oncological endpoint [30]. In our population, the low rate of residual disease following minimally invasive complete thymectomy may be the result of the strict patient selection. However, the improved ergonomic characteristics of the robotic approach over thoracoscopy may enhance radicality and may be taken into account in the evaluation of minimally invasive surgery for larger thymomas or thymic carcinomas.

Our study has some limitations. One is its retrospective nature, which includes differences in the number of robotic versus thoracoscopic procedures; this variation may imply risk of bias. Hopefully, these risks have been mitigated by the use of a propensity score matched analysis, comparing two study groups with more homogeneous clinical, demographic and surgical characteristics. Secondly, the ergonomic score adopted in this report has not been formally validated; although, so far, no previously validated scoring systems based on ergonomic features were available. In addition, despite the fact the scoring system adopted in this study was based on objective findings, the scores were still assigned by surgeons, resulting in risks of subjectivity-related bias. To overcome this limitation, a recent study has suggested the usefulness of artificial intelligence-based assessment for surgical procedures [31]. In future studies, the implementation of artificial intelligence with scoring systems based on objective findings might help clarify differences in ergonomic characteristics between robotic versus thoracoscopic approaches in a reproducible, objective manner. A further limitation relates to the multicentric design of the study, which may have led to further heterogeneity and bias, although all involved centers disclosed similar long-term experience in minimally invasive thymectomy. Finally, in this study, only early-stage thymomas were enrolled; although, in nearly half of the enrolled population, the tumor dimension was larger than 4 cm, suggesting the reliability of our results, even in the presence of relatively large thymomas.

## 5. Conclusions

In conclusion, in this study, robotic complete thymectomy resulted in significantly higher scores in the total maneuverability domain and in fewer intraoperative adverse events compared to thoracoscopic surgery. On the other hand, no intergroup difference in operative morbidity and residual disease was found.

Overall, this study has allowed us to express in a quantitative manner, a previously suggested ergonomic advantage of the robotic surgical approach when dealing with excision of thymic tumors. Prospective studies are now warranted to confirm our encouraging preliminary findings.

## Figures and Tables

**Table 1:** Scoring system employed for the ergonomic domain assessment.

**Maneuvering**	
The accuracy of surgical maneuvers during isolation of target structures	
3	Optimal perception of depth, target tissue reached with no missed movement
2	Good perception of depth with any missed movement to reach the target
1	Any intraoperative surgery-related complication
**Exposure**	
The overall visualization of the target achieved by the adopted technology	
3	Target visible and reachable
2	Target visible and reachable with minor limitations (need to change the camera or the instruments position one time per step)
1	Target visible and reachable with limitations (need to change the camera and/or instruments position two or more times per step)
**Instrumentation**	
The easiness by which instruments could be employed simultaneously without disturbing surgical vision	
3	Optimal convergence of instruments with no crowding and no need of lens cleaning
2	Moderate instrument crowding and/or need to clean the lens one time per step
1	Lack of multiple instrument convergence with fencing and/or need to clean the camera two or more times per step

**Table 2:** Demographic and perioperative characteristics in the study groups.

	Entire Population (n = 219)			Anterior Mediastinal Tumors after Propensity-Score-Match Cohort (n = 102)		
	Robotic (n = 149)	Thoracoscopic (n = 70)	*p*-Value	Robotic (n = 68)	Thoracoscopic (n = 34)	*p*-Value
Male sex, n (%)	75 (50.3)	25 (35.7)	0.06	26 (38.2)	15 (44.1)	0.568
Age, median (IQR)	63 (46–70)	59.5 (50–68.8)	0.56	62 (49.75–68)	60 (47.25–73.5)	0.952
BMI, median (IQR)	23 (21–24)	23 (22–24)	0.266	23 (21–24)	22 (22–23)	0.329
Myasthenia Gravis, n (%)	53 (35.6)	30 (42.9)	0.871	17 (25)	8 (23.5)	0.529
Left-side approach, n (%)	111 (74.5)	38 (54.3)	0.277	51 (75)	22 (64.7)	0.107
Tumor diameter (mm), median (IQR)	40 (28–50)	45 (31.5–63.5)	0.166	40 (30–54.75)	41 (30–58)	0.834
Anterior Mediastinal Tumors, n (%)	105 (70.5)	40 (57.1)	0.07	-	-	-
Operative time (min), median (IQR)	150.5 (107–180)	125 (90–180)	0.1	144.5 (103.5–171)	128.5 (105–170)	0.68
Thymic Epithelial Tumors, n (%)	86 (57.7)	34 (48.6)	0.24	63 (92.6)	26 (76.5)	0.05
Thymoma A	20 (23.3)	5 (14.7)		11 (16.2)	4 (11.7)	
Thymoma AB	26 (30.2)	11 (32.3)		22 (32.3)	9 (26.6)	
Thymoma B1	11 (12.8)	4 (11.8)		10 (14.7)	4 (11.7)	
Thymoma B2	17 (19.8)	7 (20.6)		12 (17.6)	4 (11.7)	
Thymoma B3	7 (8.1)	4 (11.8)		4 (5.9)	3 (8.9)	
Thymic Carcinoma	5 (5.8)	3(8.8)		4 (5.9)	2 (5.9)	
Thymic Cysts	10 (6.7)	9 (12.9)	0.27	-	-	-
Thymic Hyperplasia	43 (28.9)	22 (31.4)	0.15	-	-	
Other *	10 (6.7)	14 (20)	<0.001	5 (7.4)	8 (23.5)	0.05
Post-operative complications, n (%)	12 (8.1)	4 (5.7)	0.73	8 (11.8)	2 (5.9)	0.55
Arrythmias	3 (25)	1 (25)		1 (12.5)	0	
Infective complications	2 (16.7)	1 (25)		1 (12.5)	1 (50)	
Chylothorax	1 (8.3)	1 (25)		1 (12.5)	1 (50)	
Other **	6 (50)	1 (25)		5 (62.5)	0	

* Other includes: metastases, thymolipoma, primary mediastinal large cell lymphoma, large cell neuroendocrine carcinoma of the mediastinum. ** Other includes phrenic nerve palsy, haemoptysis, pleural effusion and myasthenic crisis. (BMI: body mass index).

**Table 3:** Ergonomic analysis results in both the entire population and in propensity score matched population.

	Anterior Mediastinal Tumors after Propensity-Score-Match Cohort (n = 102)		
	Robotic (n = 68)	Thoracoscopic (n = 34)	*p*-Value
**Maneuvering**			
Total	11 (11–12)	11 (9.25–12)	0.039
Lower horn dissection	3 (3–3)	3 (3–3)	1
Upper horn dissection	3 (3–3)	3 (2.5–3)	0.505
Vascular dissection	3 (3–3)	3 (3–3)	0.222
Peri-thymic fat dissection	3 (3–3)	2 (2–3)	0.003
**Exposure**			
Total	11 (10–12)	12 (9.25–12)	0.943
Lower horn dissection	3 (2–3)	3 (3–3)	0.217
Upper horn dissection	3 (2–3)	3 (2–3)	0.302
Vascular dissection	3 (3–3)	3 (3–3)	0.925
Peri-thymic fat dissection	3 (3–3)	3 (2–3)	0.027
**Instrumentation**			
Total	11 (10.75–12)	12 (10–12)	0.970
Lower horn dissection	3 (3–3)	3 (2–3)	0.175
Upper horn dissection	3 (3–3)	3 (3–3)	0.564
Vascular dissection	3 (2.75–3)	3 (2–3)	0.391
Peri-thymic fat dissection	3 (2–3)	3 (3–3)	0.424
**Total ergonomic score**	33 (31.5–36)	34.5 (29–36)	0.330

## Data Availability

The data underlying this article will be shared on reasonable request to the corresponding author.

## Supplementary Materials

The following supporting information can be downloaded at: https://www.mdpi.com/article/10.3390/jcm13071841/s1, Figure S1: Dot plot of pre and post propensity score; Figure S2: Consort flow chart indicating the patient selection algorithm; Figure S3: (A) Intraoperative adverse-events distribution rate in the overall population showing lower adverse-events in the robotic group. (B) Total maneuvering score in the overall population with anterior mediastinal tumors showing higher scores in the robotic group. (C) Correlation plot showing an inverse correlation between operative time and total maneuvering score in the overall population; Table S1: Ergonomic analysis results in the entire population; Table S2: Ergonomic analysis results according to laterality in both the entire population and in propensity score matched population. Video S1: Scoring system application examples.
