# Supplementary material for: Ergonomic Assessment of Robotic versus Thoracoscopic Thymectomy
Source: J Clin Med. 2024 Mar 22;13(7):1841. doi: 10.3390/jcm13071841 (PMC11012820; doi:10.3390/jcm13071841)
Supplement: Supplementary file 1 [file jcm-13-01841-s001.zip › jcm-2828128-supplementary.pdf]

Table S1. Ergonomic analysis results in the entire population.

	Entire population (n=219)		
	**Robotic (n=149)**	**Thoracoscopic (n=70)**	**p-value**
**Manoeuvring**			
Total	11 (11 – 12)	11 (9.25 – 12)	<0.0001
Lower horn dissection	3 (3 – 3)	3 (2.25 – 3)	0.051
Upper horn dissection	3 (3 – 3)	3 (2 – 3)	0.132
Vascular dissection	3 (3 – 3)	3 (3 – 3)	0.140
Peri-thymic fat dissection	3 (3 – 3)	2 (2 – 3)	<0.0001
**Exposure**			
Total	11 (11 – 12)	11 (10 – 12)	0.069
Lower horn dissection	3 (2 – 3)	3 (3 – 3)	0.772
Upper horn dissection	3 (3 – 3)	3 (2 – 3)	0.001
Vascular dissection	3 (3 – 3)	3 (3 – 3)	0.437
Peri-thymic fat dissection	3 (3 – 3)	3 (2 – 3)	0.015
**Instrumentation**			
Total	11 (11 – 12)	11 (10 – 12)	0.084
Lower horn dissection	3 (3 – 3)	3 (2 – 3)	0.064
Upper horn dissection	3 (3 – 3)	3 (2 – 3)	0.092
Vascular dissection	3 (3 – 3)	3 (2 – 3)	0.058
Peri-thymic fat dissection	3 (2 – 3)	3 (2 – 3)	0.420
**Total ergonomic score**	33 (32 – 36)	32.5 (29 – 36)	0.004

Table S2. Ergonomic analysis results according to laterality in both the entire population and in propensity score matched population.

	Entire population (n=219)			Anterior mediastinal tumours after propensity-score-match cohort (n=102)		
	**Left-sided (n=151)**	**Right-sided (n=68)**	**p-value**	**Left-sided (n=73)**	**Right-sided (n=29)**	**p-value**
**Manoeuvring**						
Total	11 (10 – 12)	11 (10 – 12)	0.709	11 (10 – 12)	11 (10 – 12)	0.713
Lower horn dissection	3 (3 – 3)	3 (3 – 3)	0.874	3 (3 – 3)	3 (3 – 3)	0.670
Upper horn dissection	3 (3 – 3)	3 (3 – 3)	0.439	3 (3 – 3)	3 (3 – 3)	0.677
Vascular dissection	3 (3 – 3)	3 (3 – 3)	0.239	3 (3 – 3)	3 (2 – 3)	0.142
Peri-thymic fat dissection	3 (2 – 3)	2 (3 – 3)	0.560	3 (2 – 3)	2 (2 – 3)	0.990
**Exposure**						
Total	11 (10 – 12)	11 (10 – 12)	0.641	11 (10 – 12)	11 (9 – 12)	0.601
Lower horn dissection	3 (2 – 3)	3 (3 – 3)	0.715	3 (3 – 3)	3 (2 – 3)	0.861
Upper horn dissection	3 (2 – 3)	3 (2 – 3)	0.486	3 (2 – 3)	3 (2 – 3)	0.489
Vascular dissection	3 (3 – 3)	3 (3 – 3)	0.664	3 (3 – 3)	3 (3 – 3)	0.613
Peri-thymic fat dissection	3 (2 – 3)	3 (3 – 3)	0.471	3 (3 – 3)	3 (2 – 3)	0.488
**Instrumentation**						
Total	11 (10 – 12)	11 (10 – 12)	0.968	12 (10 – 12)	11 (10 – 12)	0.416
Lower horn dissection	3 (3 – 3)	3 (3 – 3)	0.784	3 (3 – 3)	3 (3 – 3)	0.796
Upper horn dissection	3 (3 – 3)	3 (2 – 3)	0.495	3 (3 – 3)	3 (2 – 3)	0.352
Vascular dissection	3 (3 – 3)	3 (2.75 – 3)	0.934	3 (3 – 3)	3 (2 – 3)	0.194
Peri-thymic fat dissection	3 (2 – 3)	3 (3 – 3)	0.200	3 (2 – 3)	3 (3 – 3)	0.762

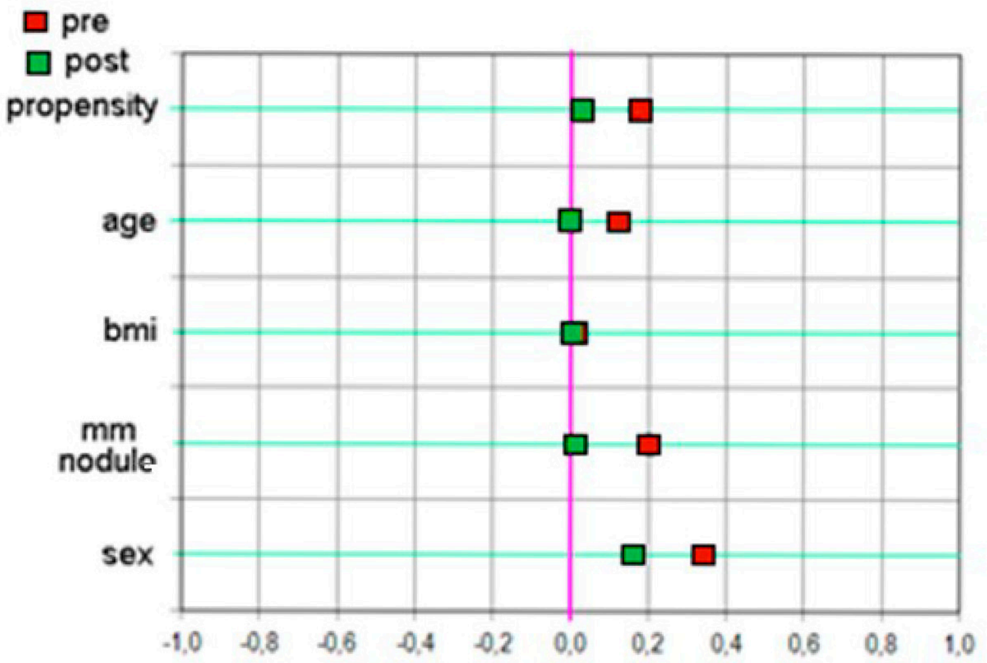

, Figure S1. Dot plot of pre and post propensity score.

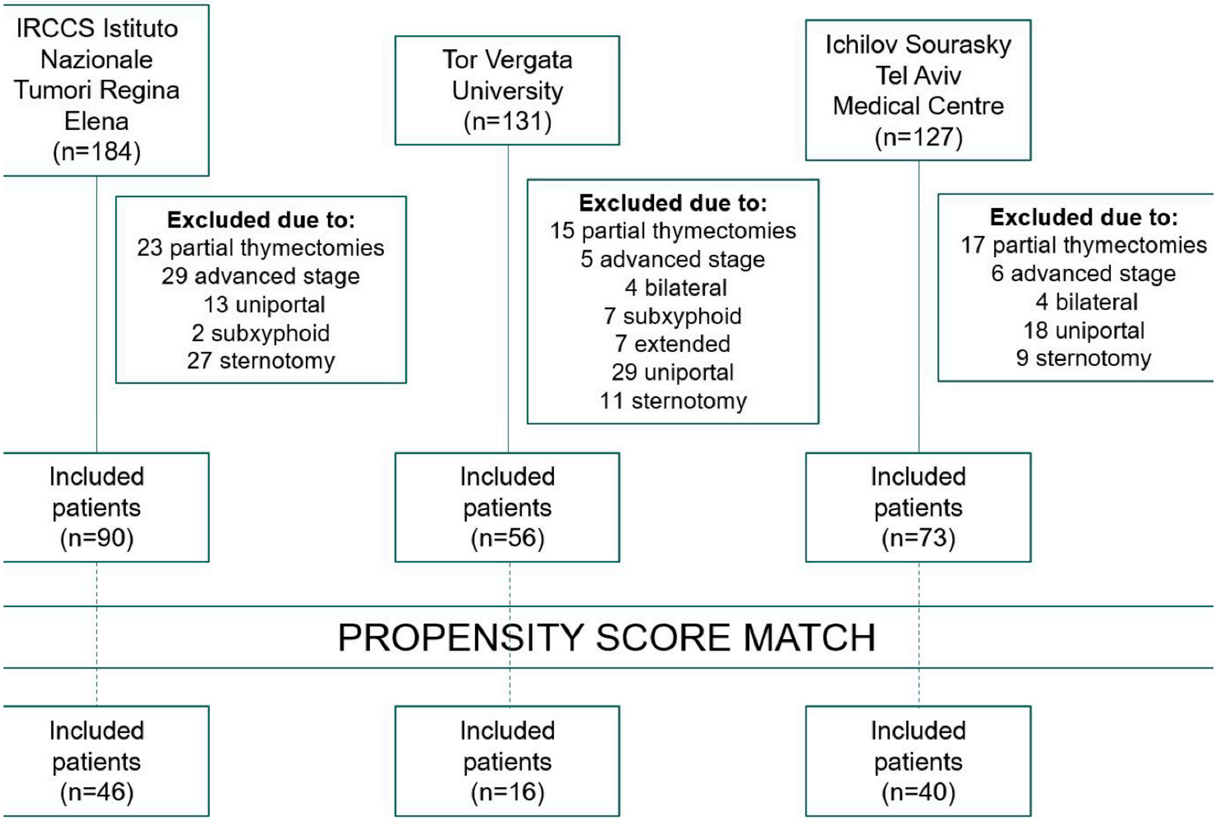

Figure S2. Consort flow chart indicating the patient selection algorithm.

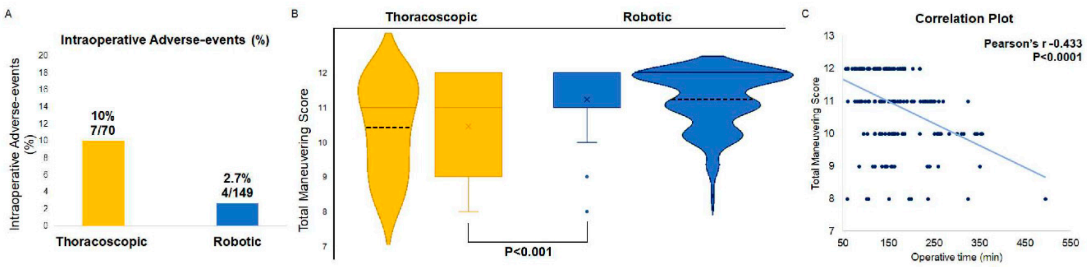

Figure S3 (A) Intraoperative adverse-events distribution rate in the overall population showing lower adverse-events in the robotic group. (B) Total maneuvering score in the overall population with anterior mediastinal tumors showing higher scores in the robotic group. (C) Correlation plot showing an inverse correlation between operative time and total ma-neuvering score in the overall population
